# Effects of Arm Weight Support Training to Promote Recovery of Upper Limb Function for Subacute Patients after Stroke with Different Levels of Arm Impairments

**DOI:** 10.1155/2016/9346374

**Published:** 2016-07-19

**Authors:** Irene H. L. Chan, Kenneth N. K. Fong, Dora Y. L. Chan, Apple Q. L. Wang, Eddy K. N. Cheng, Pinky H. Y. Chau, Kathy K. Y. Chow, Hobby K. Y. Cheung

**Affiliations:** ^1^Occupational Therapy Department, Kowloon Hospital, Hospital Authority, Kowloon, Hong Kong; ^2^Department of Rehabilitation Sciences, The Hong Kong Polytechnic University, Kowloon, Hong Kong; ^3^Rehabilitation Department, Kowloon Hospital, Hospital Authority, Kowloon, Hong Kong

## Abstract

*Purpose.* The goal of this study was to investigate the effects of arm weight support training using the ArmeoSpring for subacute patients after stroke with different levels of hemiplegic arm impairments.* Methods.* 48 inpatients with subacute stroke, stratified into 3 groups from mild to severe upper extremity impairment, were engaged in ArmeoSpring training for 45 minutes daily, 5 days per week for 3 weeks, in addition to conventional rehabilitation. Evaluations were conducted at three measurement occasions: immediately before training (T1); immediately after training (T2); and at a 3-week follow-up (T3) by a blind rater.* Results.* Shoulder flexion active range of motion, Upper Extremity Scores in the Fugl-Meyer Assessment (FMA), and Vertical Catch had the greatest differences in gain scores for patients between severe and moderate impairments, whereas FMA Hand Scores had significant differences in gain scores between moderate and mild impairments. There was no significant change in muscle tone or hand-path ratios between T1, T2, and T3 within the groups.* Conclusion.* Arm weight support training is beneficial for subacute stroke patients with moderate to severe arm impairments, especially to improve vertical control such as shoulder flexion, and there were no adverse effects in muscle tone.

## 1. Introduction

Robots are one of the major technological revolutions in the past decade in rehabilitation training approaches for arm recovery. Robotic therapy stimulates active, assistive paretic limb movement in a reliable, controllable, repeatable, quantifiable, and flexible way that makes it an ideal tool to evaluate kinematic and kinetic measurements, implement rehabilitation paradigms, and facilitate motor recovery from stroke and other neurological diseases [1]. One of the great advantages of robots is that they allow for a higher dosage and/or intensity of delivery of training to take place than conventional rehabilitation therapy [1]. Robotic devices take the form of either an end-effector or an exoskeleton. In an exoskeletal system, the paretic limb is enclosed in an actuated robotic suit that conforms to the patient's limb configuration. It can capture full specification of the limb configuration and the force applied and allows forces to be measured independently at each joint. This provides valuable ordinal data for data analysis and evaluation of patients. However, a criticism of actuated upper extremity robots is that they allow patients to move with robotic actuators apart from the patient's effort and attention, which negatively affects their motor recovery [2].

The ArmeoSpring is a passive instrumented arm orthosis with a spring mechanism for adjustable arm weight support in a large 3D workspace, and it can be used as a real-time input device with its ancillary software Armeocontrol. It originated from T-WREX, which was developed by Reinkensmeyer et al. (2002) [3] to provide an additional orthosis with elastic bands to counterbalance arm weight and assist in arm movement with more degrees of freedom across a large workspace [3, 4]. Position sensors and grip sensors allow feedback on movement and grip force [3]. The ArmeoSpring is particularly useful for patients with low muscle strength, especially patients who have lost the function of or have restricted functions in their upper extremities caused by various neurological disorders.

Two randomized control trials (RCT) were conducted on the T-WREX system [5, 6]. In the first RCT, the T-WREX group had significantly higher gains in arm improvement (but not hand use), ability to do functional tasks, and reaching an increased range of motion compared to that of the control group at their 6-month follow-up [5]. The improved motor outcome was modest and functionally insignificant [5].

A single-blind RCT found that additional ArmeoSpring training led to significant improvements in shoulder adduction-abduction and normalized jerk compared with conventional training in acute stroke patients [6]. A single-group ArmeoSpring study for mild to moderate cases of hemiparesis found significant improvements in movement ability and some upper arm functions at 12-week and 4-month follow-ups [7]. Although studies show greater improvement of motor control of hemiplegic arms after application of robotic therapy, so far, there are no studies on whether ArmeoSpring different training modules are beneficial and whether ArmeoSpring is beneficial for people with different levels of arm impairments after stroke. The conclusion of a review in technology for supporting upper limb training after stroke also highlights the importance of future trials include outcome assessments to give evidence for the influence of technology-supported training on arm-hand function, patients' functional levels, and participation [8]. Therefore, the aim of this study is to investigate the effects of arm weight support training using ArmeoSpring by applying it to subacute patients after stroke with different levels of hemiplegic arm impairments and to evaluate the kinetic, kinematic, and functional outcomes before training, after training, and at a 3-week follow-up.

## 2. Methods

### 2.1. Participants

A total of 48 inpatients admitted consecutively to a regional rehabilitation hospital were recruited by convenience sampling. The principal inclusion criteria were (i) being diagnosed with cerebral vascular disease either by a CT scan or MRI in a medical report and compatible with unilateral hemispherical involvement; (ii) being within 1 week to 6 months after stroke; (iii) being able to understand verbal instructions and follow two-step commands according to the Mini-Mental State Examination (MMSE) [9]; (iv) exhibiting severe to mild unilateral upper limb paresis, defined as levels 1–6 in the Functional Test for the Hemiplegic Upper Extremity (FTHUE) [10] (this ranges from beginning voluntary motion of the hemiplegic shoulder and elbow to beginning to be able to combine components of strong mass flexion and strong mass extension patterns in the hand); and (v) having predominant spasticity over elbow flexion with scores on the Modified Ashworth Scale (MAS) [11] of less than Grade 3.

Participants were excluded if they (i) had significant impairment in visual acuity, visual perception, and unilateral neglect (the star cancellation subtest in the Behavioral Inattention Test ≤51) [12]; (ii) had unstable medical conditions including unstable angina, symptomatic cardiac failure, uncontrolled hypertension (>170/110 mmHg), chronic obstructive pulmonary disease, major poststroke depression, active neoplastic disease, or significant orthopedic or chronic pain; (iii) had received botulinum toxin injections prior to the study; and (iv) had previously participated in robotic therapy in the upper extremities.

Patients were categorized into three groups according to the functional levels of the FTHUE [10]. Group 1 was for patients who just began to show voluntary movement of their shoulder and elbow (i.e., severe arm impairment; functional levels 1-2), Group 2 was for patients who had a more active range of movement in their shoulder and elbow (i.e., moderate arm impairment; functional levels 3 and 4), and Group 3 was for patients who demonstrated more mass combination or isolated proximal or distal control movement (i.e., mild arm impairment; functional levels 5 and 6). Patients who fell within the exclusion criteria categories or contraindicated as recommended by the manual were not involved in the training system.

### 2.2. Interventions

**Figure 1:**
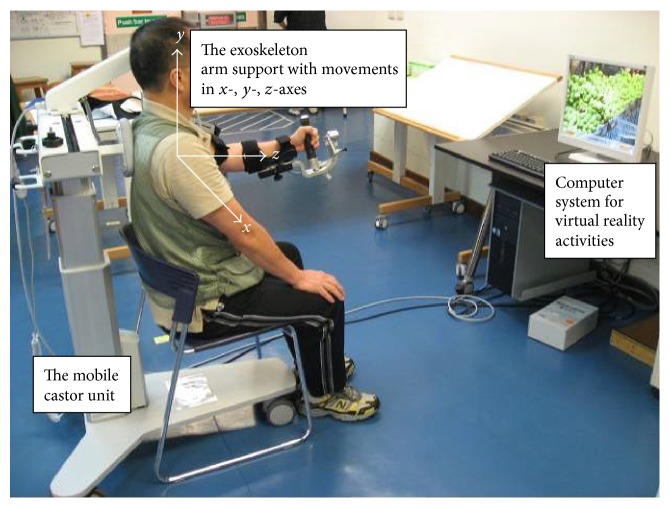
The ArmeoSpring, which was developed based on the T-WREX, is an arm exoskeleton device that combines arm gravity support with virtual reality activities.

ArmeoSpring facilitates the patient's own active movements as directed through specific virtual reality computer tasks or games that allow for self-training with immediate performance feedback (Figure 1). It is an ergonomic and adjustable arm support that counterbalances the weight of the patient's arm to enhance residual arm functions and active movement across a large three-dimensional workspace. It has a grip sensor to combine training of the hand and arm function. The built-in sensors and ancillary software can record the patient's active arm movement at each joint during the training activities.

The goal of the ArmeoSpring training modules is to teach patients to move through a smooth path with a minimum jerk trajectory with immediate feedback. All training modules were designed according to the minimum motion the participants achieved according to the upper limb functional level in the FTHUE [10]. The test was developed according to Brunnstrom's developmental stages of stoke recovery according to a hierarchy of seven functional difficulty levels, and it has been validated in Hong Kong by adding culture-specific tasks, such as using chopsticks [13]. The activities and difficulty levels were chosen to challenge the functional level of the patient's upper extremity. Because the aim of the overall training was to help the patient reach the next stage of recovery, when patients showed improvement within a functional level, therapists moved them to a more advanced training module.

**Figure 2:**
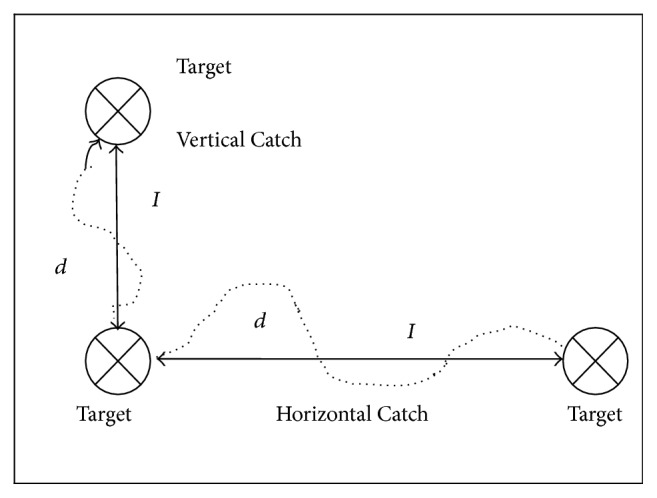
Hand-path ratio = *d*/*I*, where *d* represents the actual movement of participant (dotted line) and *I* represents the shortest steady linear movement (dark horizontal line).

The training module for Group 1 was for patients who had just started to exhibit voluntary movement of the shoulder and elbow, and the training tasks involved only one- and two-dimensional tasks at a lower level of difficulty. Training modules for Group 2 involved activities with a greater range of shoulder and elbow movement such as one- and two-dimensional activities at a higher level of difficulty and consisted of more two-dimensional tasks. The one- and two-dimensional tasks can be divided into either horizontal or vertical catching tasks (Figure 2). An example of horizontal catching is catching a moving red ball with a robotic arm in a horizontal plane, and an example of vertical catching is catching a ladybug with the robotic arm in a vertical plane. Training modules for Group 3 involved more mass combination or isolated proximal or distal control movements. The training tasks involved one-, two-, and three-dimensional tasks with a focus on three-dimensional tasks at a higher level of difficulty, for instance, forearm pronation and supination and activating the grip control sensor for grip-power training. The three-dimensional tasks included a forward reaching action by perceiving the depth of the target.

### 2.3. Procedures

This was a prospective single-group cohort study. This study was approved by the human subjects' ethics committee of the hospital. All participants signed informed consent forms. All participants received daily 45-minute sessions for the arm weight support training, 5 days per week for 3 weeks in addition to conventional rehabilitation training in the hospital, which included 60-minute activities of daily living training and affected arm horizontal and vertical reaching activities in occupational therapy, 60-minute biomechanical training in upper and lower limbs as well as gait training in physiotherapy, 30-minute speech therapy by appointment, and occasional patient and family discussions with healthcare workers at the rehabilitation hospitals.

Two ArmeoSpring devices were set up in the occupational therapy department in the hospital. Each device was designated for either left or right arms. Four occupational therapists were trained on the procedures for setting up the device on patients. After setting up the ArmeoSpring training tasks, participants were instructed to engage in training without a therapist present. To put the patient in the ArmeoSpring, the therapist has to adjust the arm orthosis to fit the patient's dimensions according to the setup procedures. Before setting up training activities, the patient has to be calibrated on the range of active movement on the computer's working plane. Once the workplace setup is complete, the therapist can select the appropriate preset training modules of activities from a wide range of functional activities in the form of virtual reality practice for the patient.

To ensure the patient's safety, an additional safety belt was designed to prevent patient falls and compensatory trunk movements during training. The device and system were checked regularly for any defects. Defects were reported to the company agent immediately. Trainings were also stopped if there was an increase in spasticity to Grade 3 or above on the Modified Ashworth Scale (MAS) [11].

### 2.4. Measurements

Evaluations were conducted, and data was collected at three measurement occasions: immediately before training (T1); immediately after completion of the three-week training (T2); and at a three-week follow-up after completion of the three-week intervention (T3), by a rater blind to participants who had received ArmeoSpring training. The primary outcome measures were (i) the Fugl-Meyer Assessment (FMA) Upper Extremity Score and Hand Score to measure arm impairments [14]; (ii) active range of motion (AROM) of shoulder flexion and shoulder abduction, elbow resting range and elbow flexion, and forearm supination and pronation; and (iii) power grip. (i) The secondary outcome measures were muscle tone of elbow as evaluated by the MAS [11] and (ii) the Functional Independence Measure (FIM) was used to measure basic functional performance (evaluated at T1 and T2 only) [15]. The FMA has 22 items measured on a 3-point scale with a maximum total score of 66. The total score can be further divided into Upper Extremity Subscore (shoulder and elbow) (max. score = 36) and hand subscores (wrist, grips, and coordination) (max. score = 30) [14].

Armeocontrol, an ancillary software program, captured secondary kinetic outcomes. They included (i) the hand-path ratio (as captured by horizontal and vertical catching levels 1–4), which indicates the extent to which the user deviates from the ideal straight line between two objects when moving from one to the next (Figure 2); (ii) the percentage of completed and time scores as captured by *x*- and *y*-axes during horizontal and vertical catching (Figure 1). *x* values represent positions of the endpoint in the lateral direction (left to right); *y* values are in the vertical direction (up to down), and *z* values correspond to the horizontal direction (far to close). All values are reported relative to the first joint (in the horizontal arm of the ArmeoSpring) where the arm orthosis is attached. A perfect linear movement has a hand-path ratio of 1. A hand-path ratio of 2 indicates that the length of the patient's hand trajectory was twice as long as the shortest line connecting the points.

After removing dropout cases, all available data were analyzed in an intention-to-treat analysis. The “last observation carried forward” (LOCF) method was used; that is, if a subject dropped out, missing values were replaced by the last assessment score of that variable. We used Pearson chi-square and one-way ANOVAs to compare baselines of categorical and continuous data, respectively. We used univariate ANOVAs to compare within-group differences in each group three measurement occasions, T1, T2, and T3, and one-way ANOVAs to compare the between-group differences in the gain scores: Gain 1 between initial assessment (T1) and assessment at the end of ArmeoSpring training (T2) and Gain 2 between T1 and assessment at 3-week follow-up (T3). We used Tukey's honestly significant difference method (HSD) for post hoc comparison to find significant differences for pairs of groups. Because seven instruments were used in measuring outcomes, a conservative level of statistical significance by the Bonferroni correction was set at *p* = 0.01 for within-group comparisons (i.e., 0.05 divided by three occasions) and *p* = 0.007 for between-group comparisons (i.e., 0.05 divided by seven instruments).

## 3. Results

Table 1 shows participant demographics and comparison of baselines in outcome measures. All participants completed training and postassessment but there were 5 dropouts at T3, that is, 3-week follow-up, because of lost contact. There were no significant differences between the three groups in baseline measures (*p* = 0.068–0.764). The only significant difference was arm impairment levels as stratified by the FTHUE for group allocation and 2 main brain lesion sites (*p* < 0.05). There were no significant differences in Vertical Catch (level 1) or Horizontal Catch (level 1) of hand-path ratio between any groups (*p* = 0.013–0.998).

Table 2 shows the results of within-groups comparison in each group at three measurement occasions and between-group comparisons of gain scores between the three groups. Results of within-group comparisons showed that Group 3 had no significant difference among the three measurement occasions except the Upper Extremity Score and the Hand Score of the FMA, time score of Vertical Catch, percentage and time scores of Horizontal Catch, and FIM (*p* = 0.000–0.009). Interestingly, Group 2 had significant differences across all three measurement occasions except for percentage scores of Horizontal Catch (*p* = 0.124). Group 1 had significant improvements in all kinetic and kinematic parameters (*p* = 0.000 to 0.007) except for hand-path ratios (*p* = 0.023–0.035).

Results of between-group comparisons found that there were no significant differences in change of tone at the three measurement occasions in all groups. There were significant differences in AROM shoulder flexion in both Gain 1 (*p* < 0.001) and Gain 2 (*p* < 0.001) between the three groups. Post hoc analysis indicated that differences were between Groups 1 and 2 and Groups 1 and 3. This is consistent with the results of the catching tasks, in which there were only significant differences in Gain 1 and Gain 2 of Vertical Catch in both percentage and time scores among the three groups (*p* = 0.000-0.001), Gain 1 in the Vertical Catch of hand-path ratio only (*p* < 0.001), and Gain 2 in the FMA Upper Extremity Scores (*p* < 0.001). Regarding control of the hand, the differences in FMA Hand Scores were significant in Gain 1 (*p* = 0.006) and Gain 2 (*p* = 0.006), and post hoc analysis indicated that the differences in Hand Scores were between Groups 2 and 3 for Gain 1 and Gain 2. However, differences were found only for Gain 2 for Groups 1 and 3. Most of the differences in AROM shoulder flexion, FMA Upper Extremity Scores, and hand-path ratio (Vertical Catch) were in gain scores between Groups 1 and 2 and Groups 1 and 3, whereas significant differences in gain scores of Groups 2 and 3 were mostly found in FMA Hand Scores.

## 4. Discussion

In this study, we categorized stroke participants into three groups according to their levels of arm impairment and assigned them to three different groups of training tasks accordingly (the results justified this group assignment). This study differs from previous studies because it pioneers the triage of patients to the use of ArmeoSpring that is most effective for them. Groups 1 and 2 (who were focused on regaining proximal function) showed more proximal improvement than Group 3 (which was focused on regaining distal functions). This is important to help therapists decide which treatment is right for the patient. Moreover, we found that there was no difference in change of muscle tone among the three groups, which indicates that ArmeoSpring training does not lead to any increase in spasticity in patients with different levels of upper extremity impairment, regardless of the group the participants belong to.

Within-group comparison results indicated that Group 3 did not benefit from ArmeoSpring training except for an increase in Horizontal Catch time and percentage scores. It should be noted that most of the significant differences in gain scores of outcome measures of shoulder range and proximal control were between Groups 1 and 2 and Groups 1 and 3, and there was no significant change in elbow or forearm gain scores. Across the three groups, there were more improvements in shoulder flexion than in the elbow and forearm. Group 1 showed the most improvement. These patients were mostly in functional level 2 (mean 1.9, SD 0.2) and only able to activate their shoulder movement to activate the device. Group 1 patients—those who had just started to show voluntary movement of their elbow and shoulder—demonstrated the most improvements in proximal control. Meanwhile, Group 2 patients—those with a more active range of motion in their elbow and shoulder—had the most improvements in hand control. This is because, prior to using the ArmeoSpring, Group 1 patients had significant difficulty lifting the affected arm against gravity but were able to move with the nonweight support provided by the ArmeoSpring. Another option for those patients who are not sufficient to properly use the device is training by using the ArmeoPower, a powered exoskeleton with an intelligent arm supported in a 3D space. A recent study showed that it is useful to improve upper limb motor function recovery according to the results of both ArmeoPower kinematic parameters and cortical excitability of primary motor areas in response to transcranial magnetic stimulation [16].

Moreover, in our study, Vertical Catch movement improved significantly compared to Horizontal Catch movement in terms of both percentage and time scores of Vertical/Horizontal Catch in groups of severe and moderate impairments, respectively. The results were consistent with that of the T-WREX study, which showed a trend toward larger improvements at the shoulder and elbow compared to the forearm and wrist after T-WREX training, whereas the control group had larger but no significant improvement in the forearm [6]. Other studies have reported short-term improvements in the proximal shoulder and elbow and no increase in motor control or functional abilities in the wrist or hand motor control [17–19]. The overall mechanical design of ArmeoSpring's exoskeleton and training modules is geared toward increasing vertical movement by facilitating movement of the shoulder against gravity. There is a discrepancy between functional relevance of the tasks (especially forearm pronation or supination) that is instructed and the actual movement that is performed [8]. However, this should not overshadow the significant within-groups differences made in Group 3 (patients with mild arm impairment). Group 3 showed improvements in horizontal movement scores and time.

It is surprising to find that there were no within-groups changes in hand-path ratios between the three measurement occasions in all groups. This is particularly interesting because hand-path ratios are commonly used to measure kinematic outcomes to evaluate the movement efficiency of robotic devices performing a smooth movement trajectory in virtual rehabilitation, with the shortest trajectory to the target as measure of efficient movement [20]. Our results lead us to believe that hand-path ratios may not able to reflect true neurological recovery as shown by patients. Although optimal movement can be attained with lowest energy expenditure of the upper limb, it requires the dynamic interaction of the arm and forearm and coordination of agonist/antagonist cocontraction without compensatory movement of the trunk and shoulder which is difficult to achieve [20].

Although there were obvious changes in shoulder control, hand coordination, and power grip, there were no significant differences in gain scores in overall functions as measured by the FIM among the three groups of different arm impairment levels despite different improvements in their arms. The overall improvement in arm functionality may be due to effects of conventional training or spontaneous recovery. This implies a common phenomenon of robotic therapy in which the training is able to reduce arm impairments but cannot improve overall functions. Reviews by both Kwakkel et al. [21] and Prange et al. [22] found that robotic therapy of the proximal upper limb improves short- and long-term motor control of the paretic shoulder and elbow in subacute and chronic patients but has no consistent influence on functional abilities. Both studies confirmed the potential for robot-assisted devices to improve proximal upper limb functions more than conventional therapy but could not substantiate improvement in terms of self-care functions.

Although Huang and Krakauer [1] concluded in their paper that rehabilitation efforts should focus on restoring arm functions and avoiding premature emphasis on compensation in acute and subacute stages of neurological recovery, we conclude that both restoration and compensation have to be delivered in parallel at an early stage. To reduce arm impairment and further improve arm functions, it is essential to supplement use of the ArmeoSpring with supervised training with occupational therapists in missing components of activities of daily living.

This study is subject to several limitations. First, the FIM scores were retrieved from the hospital centralized case management system and the data at T3 were not known. There was also no control group in this study and that the results were not compared with parallel studies highlighting effects on arm control and muscle tone of other robotic-based protocols. Different training protocols including frequency and duration of training regimens should be considered to maximize treatment effects. In the training modules, there were fewer games with forearm tasks, which might have placed Group 1 at a disadvantage because they may be physically unable to participate.

## 5. Conclusion

The ArmeoSpring is beneficial for subacute stroke patients with moderate to severe arm impairments particularly in improving vertical control (such as shoulder flexion). Using ArmeoSpring will not induce any adverse effects in muscle tone.

## Figures and Tables

**Table 1:** Participant demographics.

	All (*n* = 48)	Group 1 (*n* = 17)	Group 2 (*n* = 20)	Group 3 (*n* = 11)	*p*
Age	60.4 ± 14.4	56.2 ± 14.1	65.7 ± 13.5	57.3 ± 14.7	0.096
Gender, *n* (%)					0.536
Male	36 (75)	14	15	7	
Female	12 (25)	3	5	4	
Type of stroke, *n* (%)					0.753
Hemorrhage	17 (35.4)	7	7	3	
Ischemic	31 (64.6)	10	13	8	
Time after stroke (days)	29.1 ± 33.0	33.7 ± 45.1	24.7 ± 14.4	30.2 ± 37.1	0.713
Hemiplegic side, *n* (%)					0.207
Right	24 (50.0)	7	13	4	
Left	24 (50.0)	10	7	7	
Main lesion site, *n* (%)					
Basal ganglia	16 (33.3)	4	9	3	0.343
Lacunar	10 (20.8)	4	4	2	0.937
Parietal lobe	6 (12.5)	5	0	1	0.024^*∗*^
Corona radiata	5 (10.4)	2	3	0	0.414
Thalamus	4 (8.3)	0	0	4	0.001^*∗*^
Pons	2 (4.2)	1	1	0	0.727
Others	5 (10.4)	1	3	1	0.655
*Baseline measures*					
FTHUE	3.3 ± 1.4	1.9 ± 0.2	3.3 ± 0.5	5.5 ± 0.5	0.000^*∗*^
MMSE	25 ± 4.3	26.4 ± 4.2	23.4 ± 4.0	25.8 ± 4.1	0.068
FIM					
Motor	47.2 ± 13.7	44.4 ± 7.0	47.7 ± 13.0	50.8 ± 21.2	0.474
Cognitive	27.1 ± 9.0	30.6 ± 11.3	24.2 ± 6.0	26.9 ± 8.3	0.091
Total	74.3 ± 17.3	71.2 ± 9.0	71.9 ± 16.0	77.7 ± 27.5	0.586
MAS	1.2 ± 0.4	1.3 ± 0.5	1.3 ± 0.4	1.1 ± 0.3	0.547

Note: FTHUE = Functional Test for the Hemiplegic Upper Extremity; MMSE = Mini-Mental State Examination.

FIM = Functional Independence Measure; MAS = Modified Ashworth Scale; Pearson chi-square for *n* (%).

*t*-test for mean ± SD; ^*∗*^ *p* ≤ 0.05.

**Table 2:** Results of outcome measures across 3 time periods and between-groups comparison of gain scores.

Variables	Group	T1 (M ± SD)	T2 (M ± SD)	T3 (M ± SD)	*p* ^†^	Gain 1 (ΔT1/T2)	*p* ^‡^	Post hoc	Gain 2 (ΔT1/T3)	*p* ^‡^	Post hoc
FMA-UL	1	7.9 ± 4.0	18.8 ± 8.2	22.1 ± 7.7	0.000^*∗*^	10.8 ± 9.0	0.017	1, 3	14.1 ± 8.8	0.000^*∗*^	1, 2; 1, 3
	2	20.1 ± 8.0	27.4 ± 7.3	28.1 ± 7.2	0.000^*∗*^	7.3 ± 5.0			8.0 ± 4.9		
	3	29.6 ± 3.3	33.0 ± 1.3	33.4 ± 1.8	0.002^*∗*^	3.4 ± 3.5			3.7 ± 3.0		

FMA-hand	1	1.0 ± 1.7	7.2 ± 6.8	11.0 ± 8.7	0.000^*∗*^	6.2 ± 6.3	0.006^*∗*^	2, 3	10.0 ± 8.4	0.006^*∗*^	1, 3; 2, 3
	2	9.5 ± 7.6	19.8 ± 9.0	21.3 ± 9.0	0.000^*∗*^	10.3 ± 6.6			11.9 ± 6.7		
	3	24.5 ± 3.0	27.6 ± 2.3	27.7 ± 3.4	0.009^*∗*^	3.2 ± 2.5			3.3 ± 3.4		

AROM (shoulder flex.)	1	21.3 ± 19.9	81.7 ± 44.2	88.5 ± 39.1	0.000^*∗*^	60.4 ± 49.1	0.000^*∗*^	1, 2; 1, 3	67.2 ± 45.3	0.000^*∗*^	1, 2; 1, 3
	2	79.4 ± 43.6	103.9 ± 42.0	41.0 ± 9.2	0.000^*∗*^	24.5 ± 23.6			26.6 ± 22.6		
	3	120.5 ± 20.0	124.6 ± 14.9	132.3 ± 15.9	0.039	4.1 ± 16.7			11.8 ± 16.5		

AROM (shoulder abd.)	1	39.2 ± 22.6	84.2 ± 40.9	89.9 ± 41.7	0.000^*∗*^	45.0 ± 43.4	0.017		50.7 ± 43.8	0.019	
	2	86.8 ± 41.5	107.9 ± 38.5	110.3 ± 36.0	0.001^*∗*^	21.1 ± 17.8			23.5 ± 25.5		
	3	110.2 ± 31.5	122.6 ± 19.2	124.4 ± 23.5	0.221	12.4 ± 26.9			14.2 ± 36.0		

AROM (elbow flexion)	1	74.3 ± 40.49	103.9 ± 26.5	110.6 ± 29.7	0.007^*∗*^	29.6 ± 41.9	0.023		36.3 ± 48.6	0.020	
	2	119.7 ± 24.3	129.6 ± 24.0	133.4 ± 19.0	0.008^*∗*^	10.0 ± 18.7			13.6 ± 20.3		
	3	134.6 ± 10.6	134.6 ± 7.9	136.4 ± 11.8	0.621	0.0 ± 11.4			1.8 ± 11.5		

AROM (forearm sup.)	1	13.8 ± 26.1	51.5 ± 46.5	58.0 ± 39.5	0.000^*∗*^	37.7 ± 46.9	0.354		44.2 ± 41.5	0.072	
	2	61.9 ± 37.7	89.5 ± 35.1	89.0 ± 37.8	0.000^*∗*^	27.6 ± 27.5			27.1 ± 24.6		
	3	92.7 ± 17.1	111.8 ± 21.9	110.0 ± 25.6	0.015	19.1 ± 10.2			17.3 ± 19.7		

AROM (forearm pron.)	1	10.3 ± 21.8	25.0 ± 37.8	46.5 ± 34.7	0.001^*∗*^	14.7 ± 38.6	0.276		36.2 ± 35.0	0.124	
	2	36.8 ± 27.0	61.2 ± 26.5	61.0 ± 26.5	0.000^*∗*^	24.5 ± 23.3			24.3 ± 22.0		
	3	65.5 ± 14.6	73.6 ± 13.8	79.6 ± 18.2	0.083	8.2 ± 9.6			14.1 ± 24.3		

Power grip (kg)	1	0.2 ± 0.7	2.4 ± 2.9	4.2 ± 4.1	0.001^*∗*^	2.2 ± 3.1	0.008		4.0 ± 4.2	0.038	
	2	3.9 ± 5.5	9.9 ± 8.4	11.3 ± 10.0	0.000^*∗*^	6.1 ± 4.1			7.4 ± 5.5		
	3	13.1 ± 5.5	15.6 ± 7.0	15.5 ± 8.1	0.258	2.6 ± 4.7			2.4 ± 6.7		

Vertical Catch (level 1) (%, score)	1	39.4 ± 23.4	84.2 ± 24.5	88.0 ± 17.8	0.000^*∗*^	44.8 ± 31.9	0.000^*∗*^	1, 2; 1, 3	48.6 ± 25.0	0.000^*∗*^	1, 2; 1, 3
	2	73.9 ± 29.0	84.1 ± 21.7	91.2 ± 16.7	0.002^*∗*^	10.2 ± 19.5			16.9 ± 22.0		
	3	92.9 ± 12.4	99.2 ± 2.71	98.5 ± 5.1	0.193	6.3 ± 13.2			5.6 ± 13.2		

Vertical Catch (level 1) (s, time)	1	95.3 ± 16.6	55.5 ± 24.2	48.1 ± 21.5	0.000^*∗*^	−39.8 ± 26.1	0.001^*∗*^	1, 2; 1, 3	−47.3 ± 24.0	0.000^*∗*^	1, 2; 1, 3
	2	70.0 ± 22.2	51.7 ± 22.7	44.1 ± 21.4	0.000^*∗*^	−18.4 ± 14.9			−25.9 ± 15.1		
	3	51.2 ± 18.1	34.9 ± 11.1	31.6 ± 15.6	0.000^*∗*^	−16.3 ± 10.4			−19.6 ± 12.5		

Horizontal Catch (level 1) (%, score)	1	23.2 ± 14.9	51.1 ± 26.0	61.5 ± 27.7	0.000^*∗*^	27.9 ± 26.3	0.079		38.4 ± 29.4	0.002^*∗*^	1, 2
	2	57.2 ± 29.0	69.3 ± 30.3	65.2 ± 31.9	0.124	12.1 ± 19.5			8.0 ± 22.1		
	3	52.6 ± 22.0	69.7 ± 18.6	80.2 ± 14.4	0.003^*∗*^	17.2 ± 11.7			27.6 ± 23.3		

Horizontal Catch (level 1) (s, time)	1	101.2 ± 18.1	82.7 ± 21.7	75.9 ± 27.3	0.005^*∗*^	−18.5 ± 28.2	0.530		−25.8 ± 32.0	0.049	
	2	80.4 ± 24.0	69.6 ± 26.5	70.2 ± 27.7	0.010^*∗*^	−10.8 ± 16.5			−10.3 ± 16.0		
	3	84.8 ± 17.2	70.1 ± 19.4	56.3 ± 17.4	0.000^*∗*^	−14.7 ± 10.6			−28.6 ± 13.0		

Hand-path ratio (Vertical Catch-level 1)	1	1.3 ± 1.1	2.6 ± 1.6	2.5 ± 1.7	0.023	1.3 ± 1.5	0.000^*∗*^	1, 2; 1, 3	1.3 ± 2.1	0.019	
	2	2.4 ± 1.5	2.3 ± 1.4	2.4 ± 1.6	0.998	−0.2 ± 1.1			0.0 ± 1.5		
	3	1.9 ± 0.5	1.5 ± 0.3	1.6 ± 0.3	0.013	−0.4 ± 0.5			−0.4 ± 0.4		

Hand-path ratio (Horizontal Catch-level 1)	1	3.1 ± 4.3	3.9 ± 3.9	3.8 ± 3.9	0.035	2.2 ± 4.3	0.029		0.5 ± 1.3	0.231	
	2	3.6 ± 3.2	3.3 ± 3.3	3.2 ± 3.3	0.354	−0.3 ± 1.5			−0.2 ± 1.5		
	3	2.9 ± 2.4	2.5 ± 1.4	2.4 ± 1.4	0.501	−0.4 ± 2.8			−0.0 ± 1.8		

Tone (MAS)	1	1.3 ± 0.5	1.3 ± 0.5	1.3 ± 0.5	0.999	0 ± 0.4	0.999		0 ± 0.4	0.766	
	2	1.3 ± 0.4	1.3 ± 0.6	1.2 ± 0.4	0.428	0 ± 0.7			−0.1 ± 0.6		
	3	1.1 ± 0.3	1.1 ± 0.3	1.1 ± 0.3	0.999	0 ± 0.0			0 ± 0.5		

FIM	1	71.2 ± 9.0	89.5 ± 11.6	NA	0.000^*∗*^	18.4 ± 9.6	0.522		NA	NA	
	2	71.9 ± 16.0	93.6 ± 14.0	NA	0.000^*∗*^	21.8 ± 9.4			NA		
	3	77.7 ± 27.5	95.5 ± 24.3	NA	0.003^*∗*^	17.7 ± 14.9			NA		

Note: T1: outcome measures immediately before training; T2: outcome measures immediately after training; T3: outcome measures at a three-week follow-up after training; ΔT1/T2: T2 minus T1; ΔT1/T3: T3 minus T1; Group 1: severe arm impairment; Group 2: moderate arm impairment; Group 3: mild arm impairment; FMA-UL = Fugl-Meyer Assessment (FMA) Upper Extremity Score; FMA-hand = Fugl-Meyer Assessment (FMA) Hand Score; AROM = active range of motion; flex. = flexion; abd. = abduction; sup. = supination; pron. = pronation; MAS = Modified Ashworth Scale; FIM = Functional Independence Measure; Post hoc: Tukey HSD; the optimal value of hand-path ratio is 1; ^†^within-group comparison ^*∗*^ *p* ≤ 0.01; ^‡^between-groups comparison ^*∗*^ *p* ≤ 0.007.
